# Hydrogel-based therapies for diabetic foot ulcers: recent developments and clinical implications

**DOI:** 10.1093/burnst/tkae084

**Published:** 2025-02-06

**Authors:** Shuao Zhao, Xinyu Hu, Yiwen Zhao, Yige Zhang, Yesheng Jin, Fei Hua, Yong Xu, Wenge Ding

**Affiliations:** Department of Traumatic Orthopaedics, Third Affiliated Hospital of Soochow University, Soochow University, No. 185 Juqian Road, Changzhou 213003, Jiangsu, China; Orthopaedic Institute, Suzhou Medical College, Soochow University, No. 178 East Ganjiang Road, Suzhou 215000, Jiangsu, China; Department of Traumatic Orthopaedics, Third Affiliated Hospital of Soochow University, Soochow University, No. 185 Juqian Road, Changzhou 213003, Jiangsu, China; Department of Traumatic Orthopaedics, Third Affiliated Hospital of Soochow University, Soochow University, No. 185 Juqian Road, Changzhou 213003, Jiangsu, China; Department of Traumatic Orthopaedics, Third Affiliated Hospital of Soochow University, Soochow University, No. 185 Juqian Road, Changzhou 213003, Jiangsu, China; Orthopaedic Institute, Suzhou Medical College, Soochow University, No. 178 East Ganjiang Road, Suzhou 215000, Jiangsu, China; Department of Orthopaedics, The First Affiliated Hospital of Soochow University, Suzhou Medical College, Soochow University, No. 899 Pinghai Road, Suzhou 215000, Jiangsu, China; Department of Endocrine, Third Affiliated Hospital of Soochow University, No. 185 Juqian Road, Changzhou 213003, Jiangsu, China; Orthopaedic Institute, Suzhou Medical College, Soochow University, No. 178 East Ganjiang Road, Suzhou 215000, Jiangsu, China; Department of Orthopaedics, The First Affiliated Hospital of Soochow University, Suzhou Medical College, Soochow University, No. 899 Pinghai Road, Suzhou 215000, Jiangsu, China; Department of Traumatic Orthopaedics, Third Affiliated Hospital of Soochow University, Soochow University, No. 185 Juqian Road, Changzhou 213003, Jiangsu, China

**Keywords:** Diabetic foot ulcer, Hydrogel dressings, Tissue engineering

## Abstract

The diabetic foot ulcer is among the most serious diabetes-associated complications, with a long disease course considerably increasing the pain and economic burden of patients, leading to amputation and even death. High blood sugar is characteristic of diabetic foot ulcers, with insufficient blood supply, oxidative stress disorder, and high-risk bacterial infection posing great challenges for disease treatment. Advances in hydrogel dressings have shown potential for the management of diabetic foot ulcers involving multisystem lesions. This study comprehensively reviews the pathogenesis of diabetic foot ulcers and advances in hydrogel dressings in treating diabetic foot ulcers, providing innovative perspectives for assessing the nursing care requirements and associated clinical applications.

Highlights

- The etiology and management of diabetic foot conditions are summarized.
- The functional needs of hydrogels for the treatment of diabetic foot ulcers are methodically outlined, taking into account the physiological, physical, and chemical aspects.
- Recent advancements in responsive and multifunctional hydrogels for the treatment of diabetic foot ulcers are reviewed.
- The safety and efficacy data and challenges of hydrogel dressings for the treatment of diabetic foot in clinical settings are reviewed.

## Background

**Figure 1:**
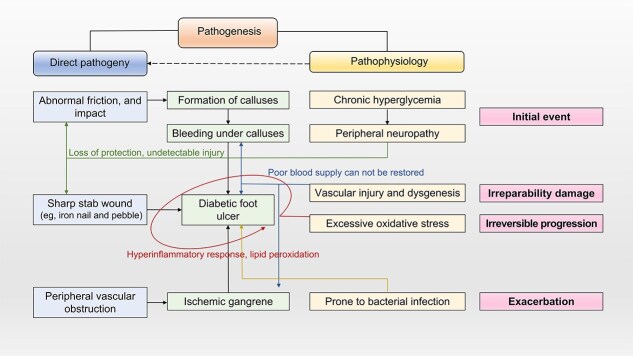
Pathogenesis of diabetic foot ulcer, including the direct causes and pathophysiological mechanisms

Diabetes is among the most common chronic diseases worldwide, which significantly threatens human well-being and leads to heavy medical burden [1, 2]. Diabetic foot ulcers are a serious diabetes-associated complication, usually arising from neuropathy, and result in the loss of protective sensation of feet and the development of biomechanical abnormalities [3]. Reportedly, the repeated abnormal friction of the foot during movements leads to callus formation and impact-induced minor trauma and inflammation can lead to subcocoon bleeding, which is gradually presented as full-layer ulcers [4]. Owing to the poor wound microenvironment in patients with diabetic foot, various conditions such as angiogenesis disorders, severe inflammatory response disorders, high reactive oxygen species (ROS) levels, and bacterial infections can occur following the wound, making natural wound healing difficult and resulting in chronic wounds (Figure 1) [5, 6]. Reportedly, >550 million adult individuals have diabetes worldwide, which is expected to rise to 1.31 billion by 2050 [7]. Furthermore, ~34% of individuals with either type 1 or type 2 diabetes develop foot ulcers during their lifetime [4], with >750 000 new cases being reported each year, affecting ~18.6 million people per year globally [8]. Notably, ~20% of patients diagnosed with diabetic foot ulcers undergo amputations, with the mortality rate of the disease reaching 50%–70% within a 5-year period [9].

Presently, comprehensive treatment with internal medicine, surgery, and rehabilitation medicine is the main clinical treatment approach for diabetic foot ulcers. Treatment with internal medicine aims to strengthen nursing while maintaining the stability of metabolism and internal environment such as blood sugar levels. Surgery mainly focuses on aspects such as debridement, anti-infection, reestablishment of blood supply, and wound closure. Furthermore, the auxiliary use of negative pressure wound treatment and local hyperbaric oxygen therapy have demonstrated good efficacy [10, 11]. However, despite achieving certain efficacy, various problems such as major trauma, long recovery and nursing cycles, and considerable physical and mental burden on patients during treatment remain persistent. These challenges warrant the need for novel therapeutic approaches for diabetic foot ulcers. For instance, tissue engineering therapy, a novel treatment method has been increasingly applied to treat diabetic foot ulcers, and many clinical studies on antibiotics-loaded bone cement have shown good local antibacterial effects [12]. Additionally, various common dressings such as hydrogels and chitosan [13], along with cell therapies utilizing stem cell therapy and epidermal growth factor, and autologous blood products such as platelet-rich plasma have been explored as treatment modalities. Among them, hydrogel-based treatments of chronic wounds such as diabetic foot ulcers have become a new research hotspot. Notably, hydrogel dressings are good drug carriers and can carry protein/peptide drugs, cells, herbs/antioxidants, and nano/microparticles to achieve antibacterial, anti-inflammatory, hemostatic, and other multifunctional effects [14]. Furthermore, they have been reported to achieve self-healing, responsiveness, and other functions [15], along with improving the drug release efficacy and broadening the applicability of materials.

**Figure 2:**
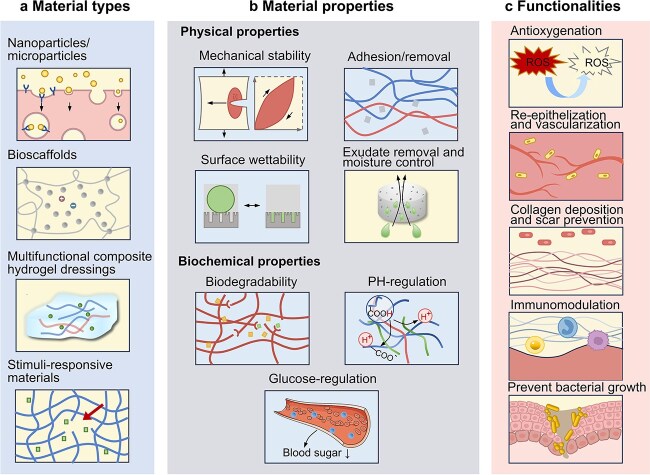
Overview of wound treatment materials. (**a**) A diverse array of materials employed in the treatment of chronic wounds. (**b**) Essential physical attributes of chronic wound care materials. (**c**) Roles of materials in wound healing

This review aims to summarize the pathophysiological characteristics of diabetic foot ulcers, common treatment modalities, and advances in hydrogel treatment approaches, including required properties and developed hydrogel dressings. Furthermore, the properties required for treating diabetic foot ulcers have been reviewed based on disease characteristics. Finally, the therapeutic advantages of existing hydrogel dressings have been evaluated to provide a theoretical basis for further developing better hydrogel dressings for treating diabetic foot ulcers (Figure 2).

## Review

### Pathophysiology of diabetic foot ulcer

#### Chronic wound features

Diabetic foot ulcers often result from peripheral neuropathy. Peripheral neuropathy in diabetics leads to decreased or loss of sensation in the foot, making the patient unable to perceive pressure, pain, and temperature changes in the foot. This loss of neural foot-protective sensation makes the foot unable to respond in time to trauma or stress and can also cause foot muscle atrophy and foot biomechanical changes, which further increases the risk of foot compression and injury [3].

After the development of peripheral neuropathy-led diabetic foot ulcer wounds, the poor wound microenvironment further exacerbates the slow healing. This persistent nonhealing wound microenvironment can be mainly attributed to elevated blood glucose levels [16, 17], inadequate oxygen supply [18, 19], heightened production of ROS [20], dysregulated inflammatory responses [21], bacterial infections [22, 23], and impeded angiogenesis [24].

The diabetic foot ulcer wound is characterized primarily by the disruption of the immune microenvironment and an exaggerated inflammatory response [25]. Wound healing encompasses physiological processes such as inflammation, proliferation, and remodeling [26, 27]. However, in the pathological healing of diabetic foot ulcer wounds, these three stages are disordered and often chaotic [28]. Owing to the reciprocal amplification between an overabundance of inflammatory cytokines and the infiltration of immune cells, a vicious cycle of chronic inflammatory response may persist in diabetic foot ulcers. Macrophages play a key role in regulating inflammation and maintaining tissue homeostasis. There exists a proinflammatory M1/anti-inflammatory M2 polarization imbalance of macrophages in diabetic foot wounds. This imbalance of immune homeostasis seriously affects the healing of diabetic wounds. By regulating the metabolism and polarization of macrophages, the inflammatory response and tissue repair process of diabetic foot ulcers can be influenced. For example, promoting the polarization of M2-type macrophages can help fight inflammation and promote wound healing. Interventions in the macrophage metabolic pathways, such as enhanced oxidative phosphorylation and fatty acid oxidation can help restore metabolic balance, reduce inflammation, and promote the healing of diabetic foot ulcers [29]. In addition, excessive inflammatory response can result in vascular endothelial damage, which further aggravates vascular diseases and tissue ischemia.

ROS is a double-edged sword [30]. The proper release of ROS can enhance the wound immunity of diabetic foot ulcers, kill bacteria, and promote wound healing [31], although high blood sugar levels in diabetic foot ulcer wounds can lead to increased inflammation [32] and lead to impaired wound healing [33].

In diabetic foot ulcers, the foot tissues are usually in a hypoxic state, and high glucose exposure interferes with the stability of hypoxia-inducible factor-1, resulting in an inability to upregulate vascular endothelial growth factor in diabetic wounds, resulting in impaired angiogenesis [34]. Meanwhile, it provides favorable conditions for the growth of anaerobic bacteria, which may exacerbate infection, thereby hindering wound healing.

In diabetic foot ulcer wounds, high blood sugar levels can promote bacterial growth in a normal wound, and the infected bacteria showcase the characteristics of biofilm growth [35]. Bacteria are protected by extracellular polymers (EPSs) of biofilms [36, 37] and are protected from penetration and destruction by conventional antimicrobials [38], which makes the treatment of infectious diabetic foot ulcers even more difficult [16].

Moreover, individuals with diabetic foot ulcers frequently exhibit vascular damage in the lower extremities, which impairs the capacity for angiogenesis in foot wound areas, resulting in reduced blood flow to the lower extremities and insufficient blood supply to the tissues. This type of chronic ischemia reduces the repair ability of foot tissues. At the same time, ischemia can promote the inflammation of foot tissues and delay wound healing. In addition, vascular diseases can cause foot microcirculation disorders, accelerating foot tissue hypoxia and metabolic waste accumulation, which then further aggravate tissue damage. Given that the feet support the entire body’s weight and are subject to considerable movement, foot injuries are more likely to tear from local motion [23, 39].

#### Bone injury features

The risk of foot bone injury in diabetic patients is far greater than that in the general population, which is often caused by neuropathy, trauma, and bone and joint disorder syndrome with metabolic abnormalities, and it is characterized by increased chances of infection and higher surgical complications, which can lead to the process of Charcot’s neuroarthropathy in severe cases and further cause joint destruction, bone loss, and deformity [40].

In diabetic patients with Charcot’s neuroarthropathy, due to the accumulation of glycoylation end-products in organic bone matrix caused by high glucose levels and bone weakening, the risk of fracture is significantly increased, with the incidence of foot fracture of up to 26% occurring within 12 months from the time of the diagnosis of Charcot’s neuroarthropathy [41]. Meanwhile, a diabetic foot is more likely to be complicated with bacterial infection, and the surgical site infection rate of diabetic patients is 13.2%, which is five times more than that of nondiabetic patients [42]. Repeated stimulation of infected tissues in lesions can result in inflammatory hyperplasia of the bone and ischemic necrosis of new bone, which seriously hinders bone regeneration. In addition, insulin resistance, secondary hyperparathyroidism due to vitamin D deficiency, bone marrow fat accumulation, and low bone turnover levels are all identified factors that contribute to the increased risk of bone injury and delayed fracture healing in diabetic patients.

### The common treatment of diabetic foot ulcer

#### Management of clinical treatment

For the treatment of diabetic foot ulcer, the main clinical methods are: (i) debridement, (ii) anti-infection, (iii) improvement of blood flow, (iv) wound closure, (v) functional reconstruction, and (vi) rehabilitation and education. Some other common auxiliary treatments include negative pressure therapy, local oxygen therapy, and hyperbaric oxygen therapy [43].

Clinical treatment principles for diabetic foot ulcers mainly include: (i) thorough debridement—the primary measure—including wound decompression, removal of necrotic and untenable tissues, and effective drainage, which directly determines the wound healing process [44]; (ii) 50% of diabetic foot ulcer wounds can result in infectious osteomyelitis [45], and, if the progression and deterioration of infection cannot be controlled, it eventually needs amputation. Early and comprehensive assessment of infections, including the categorization and grading of infection severity, is therefore essential for optimizing care for individuals with diabetic foot ulcers. Such a proactive approach is vital in averting the need for foot or limb amputations [46]. (3) Patients with diabetic foot ulcers often have peripheral artery disease, which leads to insufficient local blood supply. Presently, addressing diabetes-related wounds involves alleviating excessive foot pressure and addressing ischemia via vascular reconstruction, with the therapeutic goal of fully correcting ischemia and improving diabetes-related wound healing [47]; (4) during rehabilitation and healing, it is necessary to effectively monitor and manage the overall health of the patient, such as by reducing weight and managing diet and blood sugar levels [48]; customized orthopedic shoes are also an effective means of prevention and rehabilitation treatment [49].

In addition, the clinical characteristics of traditional Chinese medicine include infection control, wet to dry, separation of necrosis, and promotion of healing four links. These goals can be achieved with the use of traditional Chinese medicine ointment and other special treatment modes, such as the use of golden yellow cream in the nonulcer period to disperse stasis and knots or the use of Shengji Yuhong cream in the ulcer period, to lift pus and remove rot, promote the necrotic tissue to liquefy, and promote the growth of granulation and epithelial tissues.

#### Tissue engineering and regenerative medicine treatment

Various regenerative medicine treatment approaches have been reported for diabetic foot ulcers, such as common active dressings including bone cement and hyaluronic acid (HA), cell therapies including stem cell therapy, and autologous blood products such as platelet-rich plasma.

The use of bone cement as a carrier, a novel research direction, supports drugs such as antibiotics in treating diabetic foot ulcers, facilitates the development of novel materials of composite bone cement, and improves the drug loading and release efficiencies. Implanting bone cement into the wound results in the production of a 1–2 mm thick fibrous biofilm with rich blood flow [50], also called induced membrane, after 2–4 weeks. This induced membrane is a thin, translucent pseudosynovium, unlike the typical thick, white periosteum-similar structure involved in bone reconstruction [51]. Furthermore, this membrane can induce the release of various cytokines, exhibits angiogenic capabilities, and presents potential osteogenic characteristics [52, 53]. Simultaneous blocking of delta-like ligand 4/neurogenic locus notch homolog protein 1 signaling pathway promotes the vascularization of the induced membrane [54], and accelerated vascular regeneration and enhanced blood supply to ischemic flaps leads to improved wound coverage [55]. This promotes wound healing and reduces the hospitalization duration for patients [56].

In clinical settings, antibiotics-loaded bone cement is often used to treat diabetic foot ulcers. Antibiotics-loaded bone cement exhibits elution characteristics, offering notable advantages of localized antibiotic therapy and direct delivery of high concentrations of medication to the infected zone compared with that of systemic antibiotic administration [57]. This direct action effectively prevents the emergence of drug-resistant strains, exhibiting bactericidal effects owing to direct action on the diseased area, and shortens the period of bacterial transmutation within the wound [58]. In various studies, the bone cement group has demonstrated superior outcomes regarding the Numerical Rating Scale pain scores, hospitalization duration, expenses, the extent of wound reduction, healing duration, lower complication rates, and reduced infection recurrence [59, 60].

Reportedly, mesenchymal stem cells (MSCs) have shown great potential in treating diabetic foot ulcers via the promotion of angiogenesis, anti-inflammatory effects, tissue injury repair, and wound healing [61]. MSCs can carry H19 long noncoding RNA through exosomal secretion, upregulate the phosphatase and TENsin homolog deleted on chromosome 10 gene pathway, enhance the proliferation and migration ability of fibroblasts, and inhibit the apoptosis in fibroblasts [62]. Furthermore, through the mitogen-activated protein kinase (MAPK)–protein kinase B (Akt) pathway-mediated paracrine mechanism, MSCs have been shown to improve cell viability, wound healing, and antioxidant stress of Human Umbilical Vein Endothelial Cells (HUVEC) and Human Skin Fibroblasts (HSF) cells [63]. Clinical studies have shown that MSC-based treatment of diabetic foot ulcers significantly promotes ulcer healing, reduces recurrence rates, and improves amputation survival [64, 65].

Over the past few years, the use of living hydrogels as an innovative wound dressing for treating diabetic ulcers has garnered considerable interest. In addition to the inherent qualities of hydrogels, such as malleability, permeability, and biocompatibility, these advanced hydrogels have incorporated bioactive molecules, which allows them to accordingly respond to their dynamic environment and release therapeutic agents, mimicking the functions of living organisms [66]. For instance, Lu *et al*. constructed a biologically active hydrogel, namely, HP@LL_VEGF, by integrating engineered probiotics (*Lactococcus lactis*–secreting vascular endothelial growth factor [VEGF]) and temperature-sensitive hydroxypropyl methylcellulose [67]. HP@LL_VEGF exhibited a low viscosity at ambient temperatures, facilitating its application, and it rapidly transitioned into a gel state upon contact with body heat, making it suitable for topical application on the skin. Simultaneously, VEGF was persistently released from the genetically engineered *L. lactis*, stimulating angiogenesis and thus enhancing the wound healing process. Furthermore, the production of lactic acid by *L. lactis* contributed to the modulation of macrophage polarization toward the M2 phenotype, which is associated with reduced inflammation and an overall improvement in wound healing. Kang *et al*. engineered the microalga *Haematococcus pluvialis* (HEA) to fabricate the hydrogel matrix HEA@Gel [68]. This hydrogel exploited the photosynthetic activities of the green-phase HEA, which, under laser treatment, could emit oxygen into the surroundings. This property showed potential in mitigating hypoxic conditions, which are often found in chronic wounds, and fostering an oxygen-rich milieu, which is pivotal for effective wound healing. Furthermore, the red-pigmented astaxanthin within HEA can augment the ROS-scavenging activity of antioxidant enzymes and promote the M2-polarization of macrophages. This can be achieved through the discharge of astaxanthin-rich vesicles via exosomes, which plays a significant role in modulating inflammation and enhancing wound healing.

### Properties desired in hydrogels for treating diabetic foot ulcers

#### Desired biological properties (antibacterial activity)

Owing to impaired immune function, hyperglycemia, blood circulation disorders, and neuropathy, diabetic foot–associated chronic wounds provide an ideal environment for bacterial growth. The most common bacterial infection in diabetic foot ulcers is the Gram-positive *Staphylococcus aureus*, along with other microorganisms such as *Escherichia coli* and *Pseudomonas aeruginosa*. In diabetic foot ulcers, various bacteria adhere to the wound surface via adhesion proteins or polysaccharide structures, secrete extracellular polysaccharide substances to form a biofilm matrix, and further proliferate and aggregate, ultimately forming a biofilm [69].

The underlying mechanisms of biofilm formation are multidirectional and regulated by various factors including intracellular signaling molecules—such as cyclic guanosine diphosphate—quorum sensing pathway, extracellular matrix components—such as alginate and extracellular polysaccharide—surface characteristic hydrophilicity, and surface charge [70]. Bacteria-derived EPSs in biofilms protect bacteria from the external environment, including elimination by the body and medication, further increasing treatment difficulty.

**Figure 3:**
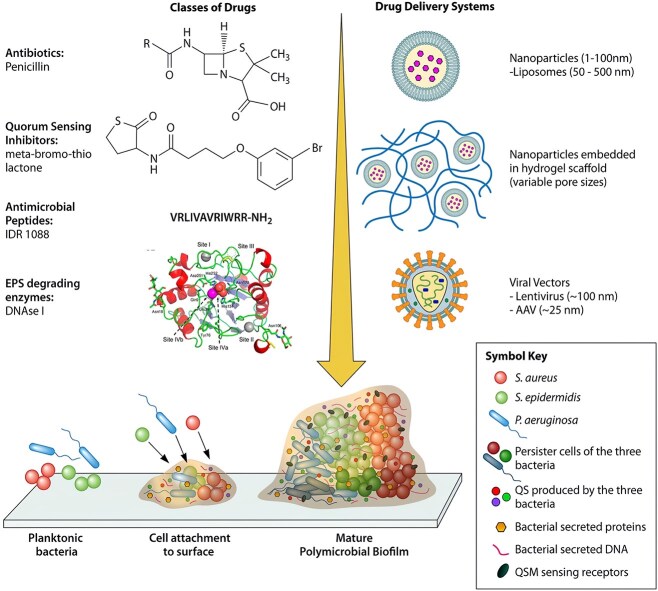
Formation of bacterial biofilms and schematics of drug delivery systems. Adapted from [69] with permission

Antibacterial hydrogels are typically classified into the following three groups based on hydrogel composition and antibacterial elements: hydrogels imbued with inorganic nanoscale particles, hydrogels integrated with antibiotic substances, and hydrogels possessing innate antimicrobial characteristics [71]. Presently, silver and zinc oxide nanoparticles are among the most prevalently utilized inorganic antimicrobial agents. Silver nanoparticles release silver ions, which inhibit bacterial DNA replication, thereby killing bacteria and exhibiting antibacterial properties [72]. Hydrogels can be combined with inherent antibacterial components to form a hydrogel skeleton. For example, quaternary ammonium salt groups, exhibiting positive charge properties, can be adsorbed to negatively charged bacterial cell membranes through electrostatic action. Subsequently, hydrophobic groups can be inserted into the lipid layer to change cell membrane permeability and destroy the membrane structure, resulting in the leakage of bacterial intracellular substances and denaturation of enzymes or proteins, thereby exhibiting the bactericidal function (Figure 3) [73].

#### Desired biological properties (pro-angiogenic ability)

Patients with diabetic foot ulcers are often accompanied by vascular injury and dysgenesis, with the incidence of lower extremity vascular lesions being as high as 45%–60%. The high glucose level and neuropathy in the blood of patients with diabetic foot ulcers cause severe dehydration of cells in blood vessel walls, degeneration, and necrosis of blood vessel endothelial cells, thereby resulting in vascular structural damage, vascular media calcification, and segmental stenosis or occlusion. This compromises the blood flow to the wounds on the feet, further exacerbating the disease and resulting in nonhealing of wounds and fractures. Therefore, the hydrogel dressing needs to have the ability of angiogenesis, especially, the formation of peripheral blood vessels to promote the healing of diabetic foot wounds.

Presently, angiogenesis-promoting hydrogels can be mainly divided based on the characteristics of their loads, as follows: hydrogels loaded with metal nanoparticles (MNPs) [74], active angiogenesis-promoting factors [75, 76], and natural plant extracts [77]. For example, MNPs can promote angiogenesis through various mechanisms. First, metal ions—certain MNPs, such as gold and copper oxide, release metal ions to stimulate angiogenesis-related cytokine production. Reportedly, metal ions activate endothelial cells and promote angiogenesis. Second, nitric oxide (NO) production—some MNPS, such as zinc oxide nanoparticles, can produce NO through the MAPK/Akt/endothelial NO synthase pathway, thereby promoting endothelial cell migration and angiogenesis. Finally, regulation of gene expression—some MNPs, such as cerium dioxide nanoparticles, can promote angiogenesis by regulating gene expression. Cerium dioxide nanoparticles stabilize hypoxia-inducible factor-1α expression within endothelial cells to promote angiogenesis [78].

#### Desired biological properties (antioxygenic property)

A dysregulated oxidative stress system in diabetic foot wounds is among the core factors leading to disease progression. Diabetic foot ulcers lead to excessive activation of the oxidative stress system owing to hyperglycemia, microvascular disease, weakening of the antioxidant defense system, and other factors. Simultaneously, the over-activation of ROS leads to excessive inflammation, endoplasmic reticulum stress, lipid peroxidation, and other lesions, which further exacerbate the disease.

Oxidative stress in diabetic foot ulcers can be generated through various mechanisms. First, hyperglycemia—diabetic foot ulcer can occur because of prolonged exposure to a hyperglycemic milieu. Chronic hyperglycemia leads to changes in the expression of advanced glycation end-products (AGEs)/receptors of AGEs and hexosamine biosynthetic pathway signaling pathways [79], thereby increasing ROS generation via the mitochondrial electron transport chain and glucose autoxification. Second, microangiopathopathy—diabetes can induce structural and functional changes in microvessels, leading to changes in local hemodynamics [80], thus affecting oxygen supply and ROS clearance. Third, weakening of the antioxidant defense system—the activity of antioxidant enzymes in patients with diabetic foot ulcers is reduced, which weakens the antioxidant defense ability, reducing effective removal of excess ROS and thus aggravating oxidative stress [81].

A disordered oxidative stress system further aggravates the deterioration of diabetic foot ulcers. ROS can induce M1-like pro-inflammatory macrophages and activate signaling pathways such as MAPK, signal transducer and activator of transcription (STAT)1, STAT6, and nuclear factor (NF)-κB, thereby promoting inflammation and interfering with macrophage differentiation [82, 83]. Additionally, ROS can directly interfere with the structural and functional integrity of proteins, lipids, and nucleic acids, resulting in cellular lipid peroxidation. The cytotoxicity of lipid peroxidation products and damage to cell membranes can further result in microvascular and macrovascular complications [84].

Liu *et al*. prepared Cu_5.4_O USNPs, which exhibited antioxidase-like activities, including simulated catalase and superoxide dismutase activities. The nanoparticles were nonsurface ligand-dependent and demonstrated high scavenging activity against three representative ROSs, namely, hydrogen peroxide, superoxide anion, and hydroxyl radical [85]. Chang *et al*. [86] inhibited the ROS-activating NF-κB pathway and found that upregulating matrix metalloproteinase (MMP)-8 and inhibiting MMP-9 enhanced diabetic wound healing. In a study, AuPt@melanin was incorporated into a hydrogel dressing (namely, GHM3), and it disrupted the ROS-driven inflammatory cycle and balanced the M1/M2 macrophage ratio, thereby alleviating diabetic rat dorsal skin and foot ulcer wounds [17]. Similarly, Rusch blue nanoparticles have been reported to emulate the functions of peroxidase, peroxidase, and superoxide dismutase, adeptly eliminating ROS [87].

#### Desired physical properties (adhesive property)

The wounds of diabetic foot ulcers are characterized by tissue ischemia, excessive inflammation, and nonmigratory epidermis, which makes natural wound healing difficult [6]. Moreover, the wound is continuously exposed to the inflammatory environment owing to the dysregulation of the inflammatory response [21], which results in persistent inflammatory exudation. Excessive exudation makes fixing the suture difficult, affecting the closure of the wound and its treatment, thereby exposing the diabetic foot ulcer wound to infection.

Presently, the main tissue adhesives used in clinical applications include cyanoacrylate adhesives and chitosan–fibrin adhesives. Cyanoacrylate adhesives exhibit good adhesion properties; however, they are difficult to degrade, may present toxic side effects, and have poor adhesion in wet environments. In contrast, chitosan–fibrin adhesives exhibit excellent biocompatibility and biodegradability; however, their adhesion to tissues is poor, making them suitable only for tissue regions with low local stress. The wounds of diabetic foot ulcers are prone to cracking due to high local stress, which necessitates the need for tissue adhesives with good biocompatibility and strong adhesion properties. Zhou *et al*. [88], exploiting the adhesive properties of snail mucus, composed a double-mesh hydrogel biomaterial using snail glycosaminoglycan and gelatin methacrylate, namely, Achatina fulica_ glycosaminoglycan (AFG)/GelMA. This hydrogel jointly promoted adhesion in tissues through chemical bonds such as amide bonds and physical contact; furthermore, it accelerated the healing of diabetic foot ulcer wounds.

#### Desired physical properties (mechanical stability properties)

Diabetic foot ulcers are accompanied by peripheral nervous system disease, affecting the responsiveness and resistance of feet to abnormal pressure, foreign entities, and foot muscle atrophy, resulting in increased skin pressure during walking [89]. The plantar pressure in patients with diabetic foot is markedly higher than that in healthy individuals [90]. A long-term uneven force on the plantar can lead to excessive keratosis of the skin and plantar callus formation, which further increases the plantar pressure. To alleviate the high-pressure environment of diabetic foot wounds, the hydrogel dressing should have good mechanical properties.

Liu *et al*. [91] prepared a double-mesh hydrogel wound dressing (namely, Gelatin-Polyacrylamide-ε-Polylysine Hydrogel (G-PAGL)) by incorporating an antibacterial chain into a polymeric matrix interlinked with polyacrylamide and gelatin (GT). Reportedly, a high crosslinking density can limit the movement of polymer networks and diminish the tensile strength of hydrogels, whereas a low crosslinking density increases the mobility within the hydrogel network, allowing it to withstand higher stretches. The volume ratio of glycerol and GT and ε-PL content was adjusted to regulate the crosslinking density and thus the mechanical properties of G-PAGL, and the toughness and ductility of G-PAGL were enhanced. Simultaneously, GT formed a second layer of cross-linked network by forming a triple helix structure, thereby augmenting the mechanical attributes of the hydrogel. The abundant noncovalent interactions in G-PAGL (including hydrogen bonding and hydrophobic interactions) could disperse external energy during deformation to protect its toughness. Altogether, owing to these mechanisms, G-PAGL exhibited high toughness, ductility, and elasticity, which ensured the functionality of its tensile and adhesion properties at −20°C–60°C. This perfectly addresses the problem of high plantar pressure regarding the mechanical properties of hydrogel for application in diabetic foot wounds.

#### Desired chemical properties (pH regulation properties)

The pH of the wound microenvironment plays an important role in the wound healing process. The pH of healthy foot skin ranges from 4.2 to 5.6, which often increases to >7 and fluctuates in the alkaline range under the influence of wound infection, hypoxia, enzymes, and other factors [92]. This alkaline environment promotes the growth of pathogenic bacteria, inhibits the function of fibroblasts and keratinocytes, and delays wound healing [93], exacerbating the wound. Hence, the regulation of the pH of the diabetic foot wound is a key part of the treatment.

In clinical practice, debridement is the predominant method for wound management. Acidic solutions can be used to adjust the pH and create a weakly acidic wound environment to combat wound infections and expedite its healing [94]. Reportedly, negative-pressure wound therapy, a noninvasive system for wound closure, has garnered widespread public attention in recent years and employs targeted negative pressure to aid in the healing of both chronic and acute wounds (Figure 2. This approach can reduce swelling, persistent discharge, and bacterial colonization, along with stimulating angiogenesis, boosting cell replication, and enhancing wound oxygenation under mechanical forces.

### The latest developments in hydrogels for treating diabetic foot

#### Treatment based on responsive hydrogel dressings (glucose responsiveness)

Glucose-reactive hydrogels can be used in diabetic foot ulcer wounds according to the characteristics of the hyperglycemic microenvironment. Hydrogel-mediated release of loaded drugs in the hyperglycemic microenvironment presents the advantages of high selectivity, sensitivity, and controllability.

**Figure 4:**
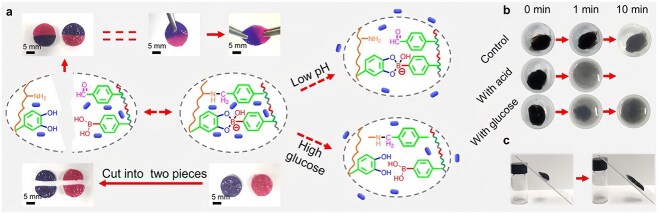
The glucose-responsive release mechanism of hydrogels: (**a**) the self-healing and triggered release properties of Polyacrylic acid-based (PC) hydrogels are contingent upon the interaction between the 1,2-diol configuration of glucose molecules and PBA. (**b**, **c**) Representative display pictures of removability. Adapted from ([99]) with permission

Presently, glucose-reactive hydrogels are mainly used to increase the glucose-responsive release ability of drugs by adding boric acid groups. Boronic acid forms stable five-membered ring complexes with 1,2- or 1,3-diols (including glucose) by coordinating its oxygen atoms with the hydroxyl group of diols. Furthermore, the complexation of boric acid and glucose is reversible, and its stability can change with changing glucose concentration changes [95]. Liu *et al*. [96] reported that phenylboronic acid (PBA) in microneedles reacted with other components (such as methacrylated polyvinyl alcohol [PVA]) to form boryl ester bonds, reversibly forming the glucose–boric acid complex. Under high glucose concentrations, boryl ester bonds break to allow the formation of a stable glucose–PBA complex, which promotes rapid drug release, such as insulin. Additionally, PBA can enhance the mechanical properties of microneedles, enhancing their compressibility and stretchability. Shah *et al*. [97] showed that PBA reacted with hydroxyl groups in quercetin, PVA, and polyethylene glycol (PEG)2000 to form a ring borate ester structure, which owing to its strong hydrophilicity, increased the carrier–water interaction, enhancing the dispersibility and dissolvability of the structure in water and thus achieving better drug release and therapeutic effects. Notably, while releasing drugs with high selectivity in a hyperglycemic environment, PBA can combine with glucose to form stable compounds, thereby reducing blood sugar levels and exhibiting potential therapeutic effects in regulating blood sugar and improving diabetes-related complications (Figure 4) [98].

#### Treatment based on responsive hydrogel dressings (pH responsiveness)

Stimulus-responsive hydrogels have been designed to change size or shape based on environmental changes (such as temperature, pH, and light). Furthermore, they can change the loaded drug release rate, showing a notable application potential regarding hydrogel wound dressings. The molecular network of pH-responsive hydrogel controlled-release systems generally contains pH-sensitive acidic or alkaline groups (such as carboxyl, sulfonic, or amino groups) or pH-response-based dynamic covalent bonds. With the changing pH value of the focal site, dissociable acidic or alkaline groups ionize, dissociating hydrogen bonds between macromolecular chain segments in the network [35] and electrostatic interactions. This results in discontinuous volume change (swelling or contraction) in the hydrogel and subsequent controlled drug release. Additionally, the dynamic covalent bonds in the system can dissociate, resulting in hydrogel degradation followed by controlled drug release.

Wang *et al*. [100] developed a highly sensitive pH-responsive intelligent hydrogel based on the biological phenomenon of “cascade amplification.” The mechanical properties of this hydrogel could be greatly changed with small changes in environmental pH. Guo *et al*. designed a multifunctional hydrogel that released metformin in a pH/glucose dual response manner for diabetes treatment. This hydrogel facilitated drug release in a mildly acidic setting, thereby promoting wound healing. Presently, hydrogels mostly contribute to wound healing under acidic conditions; however, targeted release systems for chronic wounds with alkaline pH remain lacking.

#### Treatment based on responsive hydrogel dressings (reactive oxygen species responsiveness)

In diabetic foot ulcers, excessive ROS accumulation leads to macrophage dysfunction, disrupting the normal inflammatory-to-anti-inflammatory phenotype shift. This disruption enhances inflammation and disrupts angiogenesis, thereby delaying wound healing. Hydrogels with disulfide bonds are highly sensitive to ROS and exhibit ROS-responsive behavior. Under high ROS levels, these hydrogels degrade, facilitating a controlled release of encapsulated drugs and nanoparticles, thereby regulating local ROS concentrations, facilitating the clearance of infections, and promoting wound healing.

Shi *et al*. developed an efficient nanoplatform, namely, P311@PEPS, with the potential to respond to excess ROS at the wound site and protect polypeptide P311 from enzymatic degradation. Thus, this nanoplatform effectively and rapidly promotes wound healing. Notably, polymer micelles (PEPSs) respond to excessive intracellular ROS concentrations via polypropylene thioether (PPS). Under normal conditions, a hydrophilic PEG layer covers the surface of the PEPS micelle to stabilize the micelle in the physiological environment. However, under excess ROS conditions, the PPS portion is oxidized, which increases its hydrophilicity, ultimately resulting in the disintegration of the micellar structure and thus the release of the drug-loaded P311 polypeptide [101]. Cao *et al*. [102] engineered an “explosive hydrogel” (E-gel) that responded to ROS, drawing inspiration from the sequential toxin discharge of the “exploding ant Malaysia.” This hydrogel, known as PLD/E-gel, was incorporated with small water-soluble polyhexamethy- -leneguanidine and α-lipoic acid-supported mesoporous polydopamine nanoparticles, which were encapsulated within sulfhydryl-modified HA through disulfide bonds. Shi *et al*. [103] reported a hydrogel system with ROS-responsive antioxidant properties by modifying hyperbranched polyethylene glycol diacrylate with disulfide bonds. This matrix network of this hydrogel shielded curcumin liposomes from rapid systemic clearance, thereby extending their presence at the wound site alongside silver nanoparticles. These studies show that integrating intelligent ROS reactivity, ease of injection, and multifaceted therapeutic capabilities has proven highly effective in treating chronic diabetic wounds.

#### Treatment based on multifunctional composite hydrogel dressings (loaded with nanomaterials)

Bianza *et al*. [104] fabricated an innovative composite hydrogel (namely, Reduced Graphene Oxide (rGO)-SP) by chemically reducing graphene oxide, exploiting the high electron mobility, strength, and flexibility of graphene, with teicoplanin and crosslinking with genipin in a mixture of silk protein (SP). The rGO-SP composite hydrogel exhibited considerable rGO-associated antibacterial properties, angiogenic potential, mechanical strength, and electrical conductivity for wound healing, along with the pro-angiogenic and anti-inflammatory properties of SP. Furthermore, rGO-SP exhibited high porosity, self-healing ability, shear-induced thinning, promotion of cell proliferation and migration ability, and good thermal stability. These characteristics facilitated the sustainable and long-term release of SP and rGO by rGO-SP hydrogel, along with other suitable mechanical properties. Notably, this hydrogel could effectively eradicate highly permeable bacterial biofilms on hydroxyapatite and biofilm-covered agar surfaces that mimic bone tissues. The in vivo experiments showed that the rGO-SP composite hydrogel promoted the expression of anti-inflammatory cytokines and inhibited MMP-9 activity, thereby contributing to the M2-type polarization of macrophages and angiogenesis, which significantly accelerated the healing process of uninfected and infected wounds in diabetic rats and mice, respectively. Additionally, the application of rGO-SP in infected areas of the tibia in mice has been reported to significantly promote the regeneration of bone tissue. Overall, studies on rGO-SP mixed hydrogels have shown their great potential and advantages in treating infectious diabetic wounds and preventing diabetic foot ulcers.

#### Composite hydrogel dressings (loaded with proteins/peptides)

Human type III collagen (rhCol) is an important protein in human tissues, especially in the skin and blood vessels, and plays an important role in tissue repair and regeneration, new blood vessel development, and extracellular matrix production. Collagen addition can improve the mechanical strength and stability of the hydrogel, making it more suitable as a wound dressing to provide the necessary support and protection. Additionally, the unique triple helix structure of collagen provides adhesion sites for cells; promotes cell adhesion, migration, and differentiation; and contributes to the preparation and epithelialization of the wound bed. Wang *et al*. [105] fabricated the HA-DA@rhCol complex hydrogel to promote diabetic wound healing by combining dopamine (DA)-functionalized HA with recombinant human collagen (rhCol). The HA-DA@rhCol hydrogel exhibited various optimized properties, including significantly improved *in vitro* swelling capacity, ideal degradation cycle, adjustable rheological properties, excellent adhesion, and strong antioxidant properties, along with a highly effective hemostatic effect, enhanced photothermal effect, and antibacterial action. Notably, DA addition markedly enhanced the antioxidant properties of HA and the function of inflammation regulation. This promoted the M2-polarization of macrophages, resulting in the transformation from the pro-inflammatory state to the anti-inflammatory state. Simultaneously, the recombinant human Type III collagen (rhCol) promoted angiogenesis and collagen accumulation in the wound area, accelerating tissue repair and regeneration processes in chronic wounds. Altogether, these findings suggest that HA-DA@rhCol hydrogels could promote the skin regeneration process and effectively regulate the inflammatory response pathway, showing their potential to promote the healing of chronic wounds in diabetic foot ulcer.

### Clinical aspects of developed hydrogels for treating diabetic foot

#### Clinical application advantages

In diabetic foot ulcer management, hydrogel dressings offer many therapeutic benefits over traditional wound care methods such as gauze bandages. Notably, hydrogels present notable moisture retention, drug loading capacity, sustained release of medications, functional versatility, ease of application, and contribution to enhanced patient compliance. Although traditional gauze bandages can staunch bleeding, absorb exudate, and shield wounds from infection, they cannot expedite wound healing [106, 107]. In contrast, hydrogels can foster the autolysis of necrotic tissue and maintain a moist wound environment, in addition to absorbing exudate, thereby accelerating wound healing [108]. Hydrogel dressings are superior to traditional dressings regarding their intelligent regulation of active substance release. The delivery of antibacterial, anti-inflammatory, and pro-angiogenic agents can be tailored to meet the specific needs of the healing stage of the wound, which ensures optimal treatment efficacy at different phases. Moreover, hydrogels address the issue of the initial burst release of drugs associated with traditional dressings via a controlled drug release mechanism. The preparation method of hydrogels can be refined to control the drug release profile, preventing an initial surge in drug concentration and ensuring a sustained and stable release of the medication. Additionally, hydrogels offer multifunctionality, unlike traditional dressings, which are limited to single functions such as serving as physical barriers or absorbing exudate. For instance, photoresponsive hydrogels can utilize specific light wavelengths to control drug release, allowing for noninvasive and precise spatiotemporal drug delivery deep into tissues [109]. In contrast, pH-responsive hydrogels can adjust drug release according to pH changes at the wound site, facilitating the maintenance of the physiological balance [110]. Alginate gels have high water content and expandable properties, allowing them to absorb exudate and conform to the elastic demands of various tissues. Positively charged chitosan hydrogels can rapidly promote blood clotting and stop bleeding via electrostatic interaction with negatively charged cell surfaces [111]. Hydrogels also provide the advantage of transparency, facilitating visual wound monitoring [107]. Some hydrogels possess shape memory and moldability, enabling them to be customizable to the shape of the wound for improved conformity [112]. Regarding patient compliance, hydrogel dressings can maintain a moist wound environment, which reduces the frequency of dressing changes and thus lessens the burden on patients [113]. Moreover, their ease of use allows for self-application at home, further enhancing patient compliance [13].

#### Long-term clinical safety

The US Food and Drug Administration has a total of 574 registered hydrogel wound dressings by October 2024, with numerous studies attesting to their sustained safety and efficacy. Wu *et al*. conducted a comprehensive meta-analysis including data from 278 individuals with diabetic foot ulcers across six clinical trials. They revealed that antioxidant and multifunctional hydrogels are preferable for managing diabetic wounds throughout all stages, suggesting that using diverse hydrogel types could be a potential breakthrough in treating diabetic foot ulcers, minimizing complications, and reducing associated costs [114]. Similarly, Zhang *et al*. conducted a thorough review and meta-analysis including 43 studies comparing hydrogel with nonhydrogel dressings. Reportedly, diabetic foot ulcers treated with hydrogel dressings exhibited a significantly shorter healing time (average: 7.28 days) compared with that of the control group [MD = −7.28, 95% confidence interval (CI): −11.01 to −3.55, *P* < .0001]. Moreover, the healing rate in the hydrogel group was significantly superior to that of the control group (Relative Risk (RR) = 1.57, 95% CI: 1.13–2.17, *P* = .007) [115]. Esmaeeli *et al*. showed that collagen matrix hydrogels demonstrate a markedly higher complete healing rate (60%) compared with that in the control group (35%) at the 20-week follow-up. The Kaplan–Meier survival analysis revealed the median time to complete healing for both cohorts. The median healing time for patients treated with collagen matrix hydrogel was 11.8 weeks, which was significantly less than that for the control group (21.4 weeks) (*P* = .128) [116].

#### Clinical application challenges

Various challenges limit the clinical utilization of hydrogel dressings for chronic wounds, necessitating further research for the optimization of their efficacy and safety. (i) Personalized Treatment Design: Wound characteristics and healing processes vary among patients, which necessitates selecting the appropriate hydrogel dressing based on wound size, depth, exudate volume, and nature. Hence, medical professionals need to possess advanced knowledge and skills in dressing design to apply the tailored treatment [13]. (ii) Physicochemical property balance: Hydrogel dressings must exhibit adequate mechanical strength and toughness to appropriately remain on the wound while providing good adhesion and degradability. Furthermore, optimal water absorption and moisture retention capabilities are necessary for the hydrogel to absorb wound exudate and prevent desiccation, without leading to overhydration, preventing the dressing from becoming excessively heavy or dislodging [111]. (iii) Drug release and degradation: Various factors affect the drug release and degradation in hydrogel dressings. Hydrogels containing bioactive factors need to ensure stability and delivery efficiency. For example, hydrogels containing Chinese medicine compounds present the challenge of standardization and quality control of the herbal ingredients. Additionally, photosensitive hydrogels require stable and uniform light sources with specific wavelength and intensity requirements for effective drug-release action. (iv) Patient education: It is essential for patients and their families to be well informed on the correct application and replacement of hydrogel dressings, along with the identification of signs of wound infection. This necessitates comprehensive education and guidance from healthcare professionals. (v) Cost considerations: A high cost of high-quality hydrogel dressings can potentially limit their use in certain cases, particularly in resource-constrained medical settings [13]. Therefore, the overall cost-effectiveness of the dressing must be assessed in clinical practice, including production costs, usage frequency, and replacement cycles [111]. Furthermore, hydrogel dressings may present other limitations, such as requiring specific storage conditions [113].

### Outlook

Diabetic foot ulcer is among the most serious complications in patients with diabetes. This disease has a long course and is often accompanied by refractory wounds, bone injuries, and nervous system and peripheral vascular diseases, along with a high amputation rate and mortality. However, the treatment of diabetic foot ulcers presents many challenges, including local high blood sugar levels, inadequate blood supply, oxidative stress disorders, and the risk of bacterial infection. Notably, its long course and complexity of the treatment of multi-system lesions considerably affect the health and quality of life of patients, resulting in a great social and economic burden.

Various methods have been explored for treating diabetic foot ulcers, including debridement, anti-infection, blood flow improvement, wound closure, functional reconstruction, and rehabilitation education. Reportedly, tissue engineering and regenerative medicine treatment approaches, such as bone cement, stem cell therapy, and platelet-rich plasma, play an important role in treatment. Notably, regardless of the efficacy achieved by existing clinical treatments for diabetic foot ulcers with mild conditions, the treatment is often limited to solving one or two aspects of the cause and is unable to systematically solve the root causes. This results in considerable recurrence rates of the more severe diabetic foot ulcer and long-term complications.

In recent years, owing to various advances in hydrogel dressings, as a new therapeutic means, hydrogels present the characteristics of multi-effect function and responsiveness, providing a new platform for treating diabetic foot ulcers. Herein, the systematic literature search and clinical studies revealed that antibacterial, vascularization, and antioxidant properties were crucial for hydrogels to treat diabetic foot ulcers. Furthermore, responsive hydrogel dressings should include characteristics such as glucose, pH, and ROS response to improve treatment effectiveness by regulating dynamic drug release according to the specific environment of diabetic foot ulcer wounds (such as varying blood sugar, pH, and ROS levels). Notably, hydrogel dressings are good drug carriers, and they can carry various compounds, including nano/microparticles, proteins/peptides, drugs, cells, and herbs/antioxidants, to increase the antibacterial properties, angiogenesis potential, hemostatic effect, adhesion performance, mechanical strength, and other properties of the hydrogel material to further improve its therapeutic effects on diabetic foot.

Many clinical studies have shown the efficacy of hydrogel dressings in treating diabetic foot ulcers, including shortening of the healing time, improvement of the cure rate, and good long-term safety. Although hydrogel dressings show great potential to treat diabetic foot ulcers, many challenges remain, including individualized treatment design, physicochemical properties balance, drug release control, degradation properties, patient education, and cost-effectiveness. Future studies need to focus on further optimizing these aspects to improve the overall efficacy and safety of hydrogel dressings in clinical settings.

## Conclusions

Herein, various studies on diabetic foot ulcers, including the pathogenesis of chronic wounds and multisystem lesions of diabetic foot ulcers and existing treatments, have been reviewed. Altogether, this study summarizes the corresponding treatment requirements from the aspects of biology, physics, and chemistry according to the different pathogenesis factors. Additionally, the advantages, long-term safety, and challenges of hydrogel dressings for their clinical applications were evaluated, showing the performance, potential, and progress of hydrogel dressings regarding diabetic foot ulcer treatment. Altogether, the findings of this study provide insights into the hydrogel-based treatment modalities for diabetic foot ulcers, along with a theoretical basis for the further development of better and more advanced hydrogels.
